# Development and implementation of an online clinical pathway for adult chronic kidney disease in primary care: a mixed methods study

**DOI:** 10.1186/s12911-016-0350-z

**Published:** 2016-08-17

**Authors:** Maoliosa Donald, Kerry McBrien, Wes Jackson, Braden J. Manns, Marcello Tonelli, Kathryn King-Shier, Kailash Jindal, Richard Z. Lewanczuk, Nairne Scott-Douglas, Ted Braun, Sharon E. Straus, Christopher Naugler, Meghan J. Elliott, Min Jun, Brenda R. Hemmelgarn

**Affiliations:** 1 Department of Medicine, University of Calgary, 3330 Hospital Drive NW, Calgary, AB T2N 4N1 Canada; 2 Department of Community Health Sciences, University of Calgary, 3280 Hospital Drive NW, Calgary, AB T2N 4Z6 Canada; 3 Department of Family Medicine, Cumming School of Medicine, University of Calgary, 3330 Hospital Drive NW, Calgary, AB T2N 4N1 Canada; 4 Interdisciplinary Chronic Disease Collaboration, Teaching Research and Wellness Building, GE 59, 3280 Hospital Drive NW, Calgary, AB T2N 4Z6 Canada; 5 O’Brien Institute for Public Health, Cumming School of Medicine, University of Calgary, 3280 Hospital Drive NW, Calgary, AB T2N 4Z6 Canada; 6 Faculty of Nursing, University of Calgary, 2800 University Way NW, Calgary, AB T2N 1N4 Canada; 7 Department of Medicine, University of Alberta, 8440 112 Street, Edmonton, AB T6G 2G3 Canada; 8 Li Ka Shing Knowledge Institute, St. Michael’s Hospital, 209 Victoria Street, East Building, Toronto, ON M5B 1T8 Canada; 9 Institute of Health Policy, Management and Evaluation, University of Toronto, 155 College Street, Toronto, ON M5T 3M6 Canada; 10 Department of Medicine, University of Toronto, Suite RFE 3-805, 200 Elizabeth Street, Toronto, ON M5G 2C4 Canada; 11 Cumming School of Medicine, University of Calgary, TRW Building, 3rd Floor, 3280 Hospital Drive NW, Calgary, AB T2N 4Z6 Canada

**Keywords:** Chronic Kidney Disease, Clinical pathway, Point-of-care tool, Knowledge-To-Action Cycle, Primary care, Online

## Abstract

**Background:**

Primary care physicians and other primary health care professionals from Alberta, Canada identified a clinical pathway as a potential tool to facilitate uptake of clinical practice guidelines for the diagnosis, management and referral of adults with chronic kidney disease. We describe the development and implementation of a chronic kidney disease clinical pathway (CKD-CP; www.ckdpathway.ca).

**Methods:**

The CKD-CP was developed and implemented based on the principles of the Knowledge-To-Action Cycle framework. We used a mixed methods approach to identify the usability and feasibility of the CKD-CP. This included individual interviews, an online survey and website analytics, to gather data on barriers and facilitators to use, perceived usefulness and characteristics of users. Results are reported using conventional qualitative content analysis and descriptive statistics.

**Results:**

Eighteen individual interviews were conducted with primary care physicians, nephrologists, pharmacists and nurse practitioners to identify themes reflecting both barriers and facilitators to integrating the CKD-CP into clinical practice. Themes identified included: communication, work efficiency and confidence. Of the 159 participants that completed the online survey, the majority (52 %) were first time CKD-CP users. Among those who had previously used the CKD-CP, 94 % agreed or strongly agreed that the pathway was user friendly, provided useful information and increased their knowledge and confidence in the care of patients with CKD. Between November 2014 and July 2015, the CKD-CP website had 10,710 visits, 67 % of which were new visitors. The 3 most frequently visited web pages were *home*, *diagnose* and *medical management*. Canada, Indonesia and the United States were the top 3 countries accessing the website during the 9 month period.

**Conclusions:**

An interactive, online, point-of-care tool for primary care providers can be developed and implemented to assist in the care of patients with CKD. Our findings are important for making refinements to the CKD –CP website via ongoing discussions with end-users and the development team, along with continued dissemination using multiple strategies.

**Electronic supplementary material:**

The online version of this article (doi:10.1186/s12911-016-0350-z) contains supplementary material, which is available to authorized users.

## Background

Chronic kidney disease (CKD; defined by estimated glomerular filtration rate [eGFR] < 60 ml/min/1.73 m^2^) affects 12.5 % of adults in Canada [1]. Identification and management of CKD is complex. In 2012, the Kidney Disease Improving Global Outcomes (KDIGO) Clinical Practice Guidelines for CKD were published with an aim to provide clinical practitioners with guidance for the care of patients with CKD [2]. Primary care physicians play a key role in identification, management and referral of patients with CKD, caring for approximately 95 % of patients with CKD [3]. Therefore, they are an important group upon which to focus activities aimed at dissemination and uptake of CKD guidelines. Clinical practice guidelines are intended to address evidence-practice gaps, however translating evidence into primary care can be challenging – up to 50 % of elderly patients with CKD in Alberta were not taking medications indicated to reduce cardiovascular risk and only 20 % of patients who meet referral criteria were seen by a kidney specialist [3]. Understanding this key gap, we sought the optimal knowledge translation (KT) intervention that would increase the uptake of guideline-concordant CKD care in clinical practice [4, 5].

We used the Knowledge-To-Action Cycle framework, which provides a structured approach to identifying, implementing and evaluating the uptake of evidence into practice [6]. This framework includes 7 phases: (1) identify the problem; (2) adapt knowledge to local context; (3) assess barriers and facilitators to knowledge use; (4) select, tailor and implement interventions; (5) monitor knowledge use; (6) evaluate outcomes; and (7) sustain knowledge use ([6], p.11). We used this framework to address the evidence-practice gap for CKD care (i.e. diagnosis, management and referral) in the primary care setting.

In the first three phases of this work (details of which are reported elsewhere [4]), focus groups with primary care providers and surveys of patients with CKD were followed by a stakeholder meeting with primary care physicians, nephrologists, laboratory medicine, KT specialists, researchers, patients and decision-makers. At the stakeholder meeting, the Cochrane Effective Practice and Organization of Care taxonomy framework [4] was used to ensure all relevant KT interventions were considered. At the end of this process a clinical pathway was identified as the preferred approach to facilitate CKD care in the primary care setting. In contrast to clinical practice guidelines, a clinical pathway is explicit about the timing, sequence and provision of interventions [7].

**Fig. 1:**
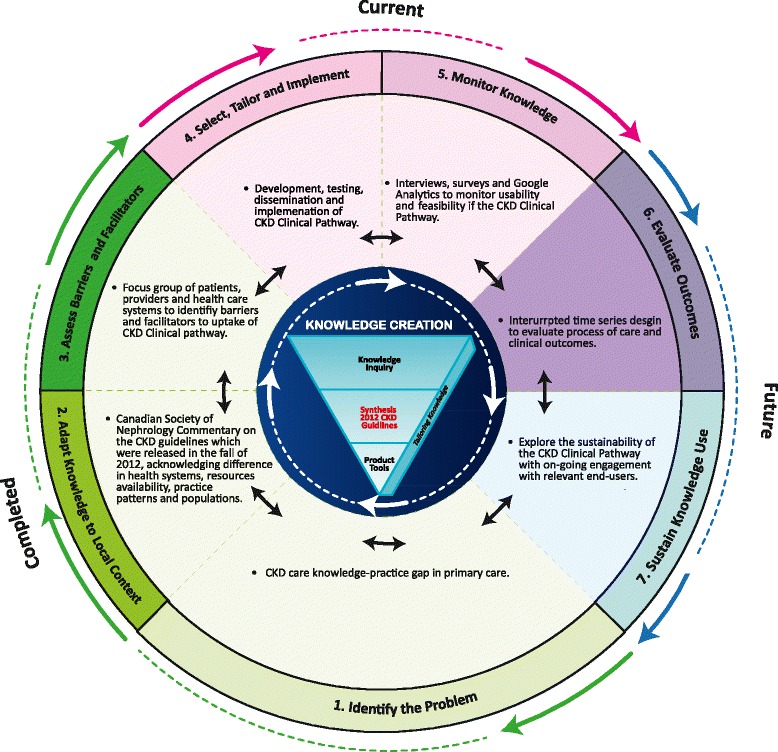
Knowledge-To-Action Cycle for the adult CKD Clinical Pathway. Adapted from Straus S, Tetroe J, Graham I. Knowledge translation in health care: moving from evidence to practice. 2nd ed. Chichester, UK: John Wiley & Sons; 2013. p.10

Given the lack of a unified definition of a “clinical pathway” [8], we considered a clinical pathway to be a “methodology for the mutual decision making and organization of care for a well-defined group of patients during a well-defined period” [9]. Clinical pathways have been shown to improve the quality, consistency and continuity of care, and enhance evidence-based and patient-focused care [10, 11]. They have also been identified by the Strategic Clinical Networks (networks of clinicians and patients with knowledge about a specific health area) at Alberta Health Services as a preferred strategy to improve the quality of care provided to Albertans [12]. We completed a scoping review to determine the availability of clinical pathways for adult CKD diagnosis, management and referral. Forty-one articles were reviewed for clinical content (i.e. screening, medical management and referral), evidence of credibility (i.e. pathway based on guidelines), format (i.e. static/interactive, electronic/paper-based) and post-implementation evaluation. The review identified a lack of detail and heterogeneity related to these elements [13]. The scoping review, together with stakeholder feedback, highlighted the need to develop a credible, evidence-based CKD clinical pathway that could be used at the point-of-care in a primary care setting. In this paper we describe phases 4 and 5 of the Knowledge-To-Action Cycle, specifically how we developed, implemented and monitored use and feasibility of the CKD Clinical Pathway (CKD-CP) (www.ckdpathway.ca) (Fig. 1). Phases 6 and 7 of the Knowledge-To-Action Cycle; evaluating process and clinical outcomes and sustaining knowledge use will be presented in a future publication.

## Methods

### Study overview

Creation and implementation of the interactive point-of-care tool (CKD-CP) for primary care providers (i.e. physicians, pharmacists and nurses involved in the care of patients with CKD) in Alberta, Canada was undertaken to assist them in three aspects of CKD care: diagnosis, management and referral.

### Development of the intervention: the CKD clinical pathway

We established a core team to develop this evidence-based intervention, including a lead investigator, project manager, knowledge translation specialists, information technology lead and representatives of stakeholder groups involved in the care of patients with CKD (i.e. primary care physicians, pharmacists, dieticians, nurses, nephrologists and local policy makers). The role of the core team was to oversee all aspects of the development and implementation of the CKD-CP. The core team worked closely with external agencies (i.e. laboratory medicine, electronic medical record vendors, primary care networks) to facilitate development in the local context. A project plan was created to guide all components of the project including responsibilities, timelines, budget, service agreements and mutual expectations. The CKD pathway development included 3 sub-phases: (1) clinic site visits and development of user profiles; (2) pathway design and usability testing; and (3) pathway deployment.

#### Clinic site visits and development of user profiles

We undertook 8 site visits and each visit took between 45 and 60 min. Interviews were conducted with 2 nephrologists, 4 primary care physicians and 2 pharmacists to document clinical processes that would impact content, use and uptake of a point-of-care clinical pathway for CKD. Our previous work with focus groups identified time constraints as a barrier to the uptake of guidelines in clinical practice [4]. Thus, by mapping each individuals clinical workflow (i.e. documenting their task sequence to diagnosing, management and referral for a patient with CKD), we were able to identify performance features of the pathway that would facilitate its efficient integration as a point-of-care tool in clinical practice. We determined that both paper-based and electronic medical records were used, sometimes in parallel to support different tasks, although the majority of health records were electronic. Also, clinicians reported varied levels of computer experience and use of online clinical tools. We identified key website features: interactive and static pages; downloadable documents; accessibility; credibility; and multi-layer content. These features are consistent with those identified by Cook and colleagues to support point-of-care resources and learning [14]. These key features and workflow data informed the design of the CKD-CP.

#### Design and usability testing

**Fig. 2:**
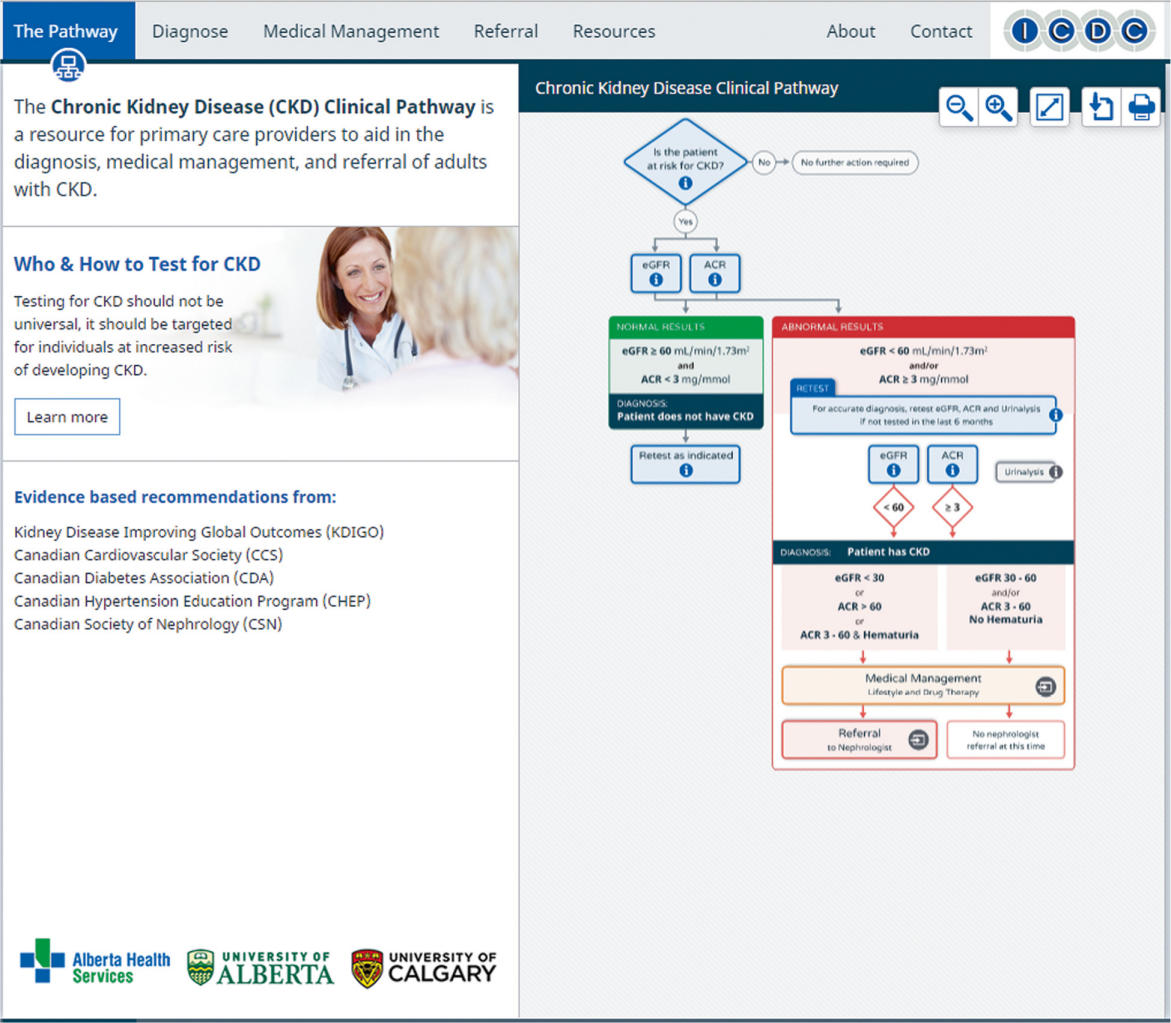
CKD Clinical Pathway home page

Based on clinician behaviors identified at the clinic site visits we created 4 user profiles (high-tech primary care physician, low-tech primary care physician, pharmacist and nurse) to capture user needs, goals, motivations, priorities and scenarios of using an online based tool. We contracted a design firm to create a prototype CKD-CP. Pathway content was based on relevant published clinical practice guidelines including the KDIGO CKD Guidelines [2], harmonized with other relevant practice guidelines (i.e. Canadian Cardiovascular Society, Canadian Diabetes Association, Canadian Hypertension Education Program and Canadian Society of Nephrology) [15–18]. Design expertise for website appearance, functionality, usability and search engine optimization were provided by a design firm. Static wireframes (visual web page schematic) were initially created, followed by an interactive click-through prototype. As website development is an iterative process, and to ensure maximum usability of the clinical pathway, we did usability testing at both the static wireframe and prototype phases. Usability testing as defined by the International Organization for Standardization (ISO) standard 9241-11 is “…the extent to which a product can be used by specified users to achieve specified goals with effectiveness, efficiency and satisfaction in a specified context of use” [19]. Studies have shown that 80 % of usability issues are detected by a sample of 6 – 8 users [20]. Therefore, we conducted usability testing with domain experts (2 nephrologists) and representatives of the target audience (2 primary care physicians, 2 pharmacists and 1 nurse practitioner). Participants were first asked a short set of demographic questions and were then provided with 3 clinical scenarios that would mimic real world practice, specifically screening, diagnosing, managing and appropriate referral for CKD. Participants worked through the scenarios using the CKD-CP prototype while engaging in a “think-aloud” exercise in which the facilitator asked the subject to communicate their thought processes verbally while they performed the task [20]. The facilitator took written notes recording both verbal responses and observations. Participants also completed a System Usability Scale questionnaire to identify usability issues [21]. Descriptive statistics and thematic coding of the think-aloud exercise were used to analyze the data. Participants provided input regarding operating systems, interactive website features, ‘levels of detail’ of information and the quality of the evidence-based resources. Participants also emphasized the need for rapid diagnosis and relevant patient handouts. These performance and usability findings (categorized by importance) were used to finalize the CKD-CP prior to deployment. The CKD-CP website has five main sections: The Pathway (home page) (Fig. 2), Diagnosis, Medical Management, Referral and Resources (Additional file 1). The website has both static information, such as patient education handouts along with interactive features (diagnosis tool, Framingham Risk Score) to provide users with more information. The main features of each section are summarized in Table 1.

**Table 1:** Description of main sections on the CKD Clinical Pathway Website

Section	Description
Home Page	• Overview of interactive CKD algorithm • List of clinical practice guidelines used to inform the clinical pathway content • Who and how to test for CKD • Disclaimer
Diagnose	• Interactive diagnose tool with drop down menu for laboratory test results • Provides CKD diagnosis and recommended care (medical management, referral) • List of investigations for causes of CKD
Medical Management	• Lifestyle management • Drug therapy (prescribing information, dosage, contraindications, general information) for ACEi/ARB, statins and antiplatelet agents • Printable patient handouts
Referral	• Referral criteria • Referral form
Resources	• Classification of CKD • Prognosis and frequency of testing • Framingham cardiovascular disease risk calculator • Management of elevated serum potassium • Clinical practice guidelines • Drug references
Contact	• Email clinical or technical questions

*Abbreviations: eGFR* estimated glomerular filtration rate, *ACR* random urine albumin-creatinine ratio, *ACEi/ARB* angiotensin converting enzyme inhibitor or angiotensin receptor blocker

#### Deployment

The interactive CKD-CP was migrated to a free public website to allow access to all end-users. Documentation, application software code and technical specifications were transferred from the design firm after the website launch. Maintenance and governance agreements with our server provider (provincial health authority) were created to ensure sustainability, ongoing support and regular updates.

### Clinical Pathway dissemination and implementation

**Fig. 3:**
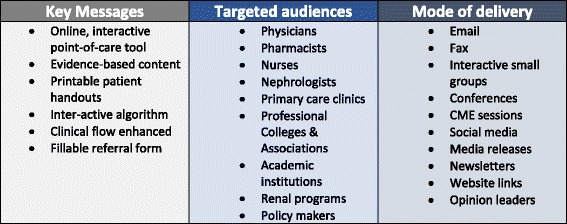
Key messages, audiences and methodology for dissemination and implementation

Our stakeholders remained engaged at all phases of the Knowledge-To-Action Cycle, including dissemination and implementation. According to Rabin et al. ([22], p.118) “*dissemination* is the active approach of spreading evidence-based interventions to the target audience using planned strategies, whereas *implementation* is the process of integrating the intervention within a setting”. Using the Knowledge Translation Planning Template –R ™ [23] we developed a multi-faceted dissemination and implementation approach to assist us in generating awareness, interest and knowledge regarding the CKD-CP and the care of patients with CKD. The strategies incorporated getting the *right information* (tailored key messages) to the *right people* (target audiences) in the *right format* (mode of delivery) at the *right time* (Fig. 3). We engaged with many organizations (i.e. professional colleges, associations), networks and academic institutions to disseminate via social media, newsletters and email. Mass media releases were done provincially. At continuing medical education events, medical trainee workshops, multi-disciplinary conferences and small group sessions we had attendees use the website as they worked through case studies. Through all our communications, we encouraged clinicians to bookmark the website for seamless access to tool.

### Monitor knowledge use

We used a mixed methods approach to monitor the CKD Clinical Pathway use and feasibility, including individual interviews to obtain users perspectives and experiences, an online survey to explore satisfaction with the tool and website analytics to capture website usage and user characteristics.

#### Individual interviews

We used a purposive sampling approach with research team members identifying potential participants (e.g. primary care physicians, nurses, pharmacists and nephrologists) to participate in in-person or telephone interviews. Semi-structured interviews were conducted 6 months after the website launch, by an experienced interviewer. Interviews were continued until saturation of themes (i.e. no new data generated) was achieved (18 participants). The interviews focused on the CKD-CPs: (1) impact on clinical workflow; (2) usability; and (3) influence on practice behaviors. The interviews, approximately 20 min in duration, were audio-recorded and transcribed. Conventional qualitative content analysis was used to interpret the interview data [24]. MD, PL and JP read entire transcripts, identified codes and sorted related codes into themes. The interview results were used to inform the content of the online survey.

#### Online survey

An online survey was used to explore satisfaction with the CKD-CP in a larger group of CKD-CP users, including clinicians and others (i.e. non-healthcare users). Primary care physicians across Alberta were sent a letter of invitation via fax using publically available fax numbers from the College of Physicians and Surgeons of Alberta [25], inviting them to complete the short online survey, administered using FluidSurveys [26]. To capture feedback from non-physician CKD-CP end-users (pharmacists, nurses, other clinicians, non-healthcare providers) and primary care physicians outside of Alberta, a “pop-up” request on the publicly available CKD-CP website was activated for a 6 week period. The pop-up request referred respondents to the FluidSurvey link. Descriptive analysis was used to report survey responses. The survey was composed of 16 closed-ended questions that addressed website usefulness, knowledge and confidence with identifying, managing and referral of patients with CKD. Participant demographic data was also collected.

#### Website analytics

We used Google Analytics, a web analytics program, to gather data on website utilization, specifically website traffic (number and type of visitors), traffic sources (geographic and internet) and visitor behaviors (content interest) [27]. Descriptive statistics were used to describe these data points captured by Google Analytics from November 2014 to July 2015.

## Results

### Interviews

#### Participants

Eighteen interviews were conducted with 10 primary care physicians, 3 nephrologists, 2 pharmacists and 3 nurse practitioners. There were 10 women (56 %) and 8 men (44 %) with 89 % practicing in an urban setting. The majority of participants (56 %) had been in practice for 11–30 years. The frequency of website use reported by participants in the prior 6 months included 5 (28 %) who reported access less than 5 times, 4 (22 %) between 6 and 10 times, 6 (33 %) between 11 and 20 times and 3 (17 %) who reported using it greater than 20 times.

#### Themes

Three major themes reflecting the barriers and facilitators to integrating the CKD-CP into clinical practice were identified, and included: *communication; work efficiency*; and *confidence* (Table 2). Interviewees reported that use of the clinical pathway resulted in enhanced communication within the multi-disciplinary primary care team and between the primary care team and the specialist and/or specialty clinic. Also, the tool was perceived to provide opportunities to augment communication (i.e. through use of patient handouts) between the health care providers and their patients. Participants highlighted the need for more patient handouts to facilitate patient self-management and information related to CKD medications. Barriers to communication included the inability to complete a direct electronic referral to the nephrologist. Participants reported that having an interactive point-of-care tool accessible by other team members could improve work efficiency, but also noted that there were technical issues based on browser version and the inability of the tool to integrate with their Electronic Medical Record. Finally, participants viewed the CKD-CP website as a credible tool, which provided knowledge and training that would help facilitate behavior change in the care of patients with CKD.

**Table 2:** Main themes identified from individual interviews

Description	Facilitator/Barrier	Quotations
Communication		
Enhanced communication between primary care physician and specialist (nephrologist) and/or specialist clinic	Facilitator	“…not creating a way that our specialist colleagues tell us what they think we should be doing. But rather how can they help us, how can they help us improve, recognizing that we don’t have the same resources, etc.” (PCP03)
Enhanced communication between primary care team members (multidisciplinary team, residents)	Facilitator	“We have access to other health care practitioners, we have educators, we have specialists that are linked to our primary care network. So this will compliment that” (PCP08) “Try to maintain continuity with a lot of our patients who see our residents…good for them so the patient knows what we’re testing and things like that” (PCP02)
Enhanced communication between primary care physician/professional and patient	Facilitator	“Augment patient explanations… visuals good for patients to reinforce information……validates our plan to them (patient)” (PCP02) “Some sort of handout for patients on what sort of meds they can be on” (PHARM01) “Part of our problem as specialists is we don’t clarify or make it (CKD care) very clear to patients” (NEPH01)
Consistency in information provided	Facilitator	“We should all get on the same page… I love the fact that they’ve chosen to do eGFRs” (PCP03) “every physician is different, but this is one resource we can turn to for consistency purposes for my practice” (N02)
Inability to do electronic referral (eReferral) from within tool	Barrier	“eReferral – link EMR and referral – forces you to put the right pieces in” (PCP03)
Work efficiency		
Point-of-care tool, with single access point	Facilitator	“It’s very quick to decide if someone has CKD, it reminds me about the things I have to do…” (PCP01) “Great to have all that information in one place” (PCP03) “Easy to quickly run through it when you’re in between patients or on a visit if you want to confirm that your management is correct” (PCP07)
Team members can use tool which assists with clinic workflow	Facilitator	“Before I see a patient my medical office assistant just clicks”(PCP08)
		“Chronic disease nurse using the CKD pathway – think that would be brilliant” (PCP06)
Technical issues with browser version	Barrier	“It is a great resource and it’s a shame that we cannot access it” (N02)
Inability to link directly to EMR	Barrier	“Hoping that this CKD pathway is to be part of our EMR” (PCP08)
Confidence		
Viewed as credible site	Facilitator	“Can be comfortable saying…. from a position of knowledge” (PCP03)
Provides knowledge and training for CKD	Facilitator	“Demonstrating CKD information to student/clerks/residents - it gives them confidence” (PCP07)
Facilitates behavior change	Facilitator	“it (tool) encourages me not to screen as broadly as I was screening” (PCP04) “comfort level did not have before, it’s like a tool for identifying patients” (PCP08) “I’ve even adjusted some of my recommendations based on it (tool) to reflect more clearly what is in the Pathway” (NEPH01)

*PCP* primary care physician, *N* nurse, *NEPH* nephrologist, *PHARM* pharmacist

### Online survey

The online survey was available from June 1^st^ to July 13^th^, 2015. Overall 159 participants completed the online survey (the number of potential respondents is unknown), of whom 141 (89 %) were healthcare providers, 9 (5.5 %) were non-health care providers (i.e. person with kidney disease or person who cares for someone with kidney disease) and 9 (5.5 %) were unknown. 52 (33 %) participants reported previously using the CKD-CP, 83 (52 %) reported this was their first use, and 24 (15 %) did not report. Of the 16 healthcare respondents with prior use who fully completed the survey, 15 (94 %) *agreed* or *strongly agreed* that the pathway was user friendly and provided useful information regarding CKD. The majority (*n* = 11; 69 %) found the printable patient handouts useful. Overall the majority of respondents (81 %) reported that the tool increased their knowledge and confidence in the care (i.e. screening, diagnosis, management and referral) of patients with CKD.

### Website analytics

**Fig. 4:**
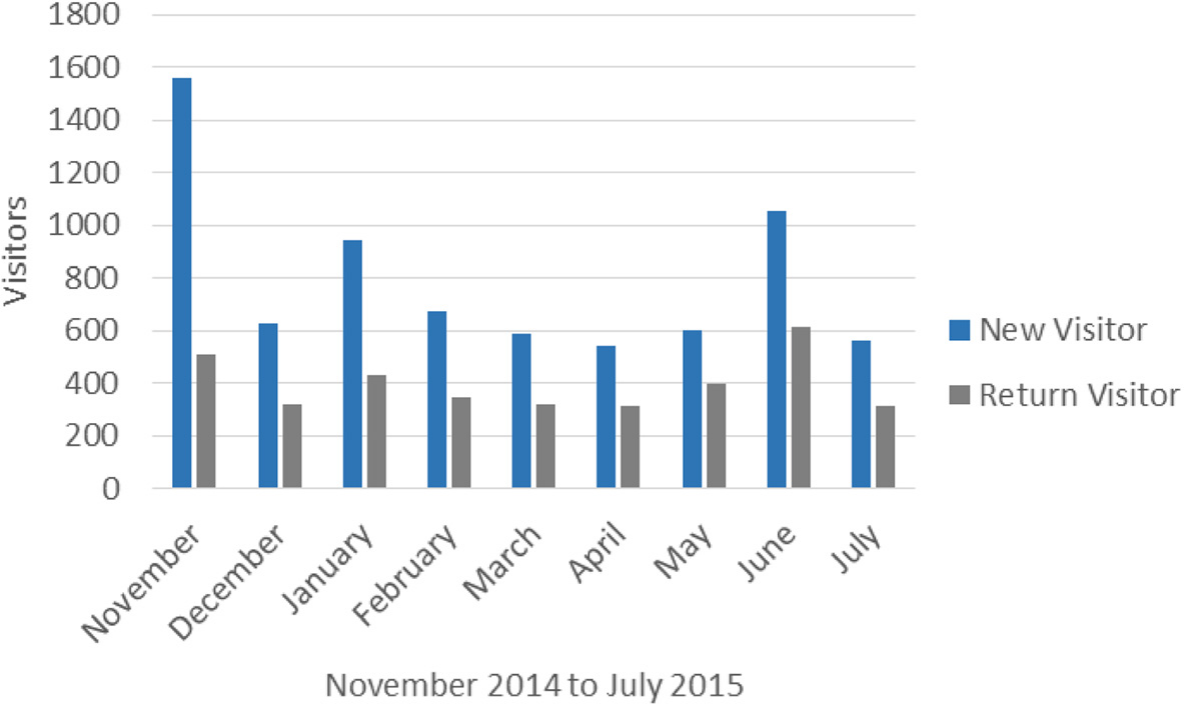
New versus returning visitors, by month since implementation

The CKD-CP was launched November 5^th^, 2014. From November 5^th^, 2014 to July 31^st^, 2015 there were 10,710 visits, with 7,146 new visitors (67 %) and 3,564 return visitors (33 %) (Fig. 4). The majority of visitors were from Canada (85 %), followed by Indonesia (6 %) and the United States (2.4 %). Fifty percent of visitors accessed the website by typing the URL into their browser or by bookmarking it as a favorite. Other users accessed it using a search engine or were referred by other professional organization’s websites. The 5 web pages most frequently viewed (average time spent in minutes) included the: *home page* (1.17); *diagnose page* (1.04); *medical management page* (2.33), *who and how to test page* (1.23); and *referral page* (1.29). There were 30,323 page views with the majority of users (42 %) starting with the *home page* and then moving to either the *diagnose page* or *medical management page*. The majority (61 %) of users leaving the *diagnose page* went to the *medical management page*. Return users are accessing the *diagnose* (4 %), *medical management* (2 %), *referral* (0.8 %) and *resources pages* (0.8 %) more often than first time users.

## Discussion

Although multiple CKD clinical pathways are available, there is significant variability in the quality, content and evaluation of the use and impact of these tools [13]. We used the Knowledge-To-Action Cycle to guide the development and implementation of an online CKD-CP for diagnosis, management and referral of adults with CKD in primary care. Indicators used to monitor and evaluate health information tools with regards to their effect and use include their reach, usefulness and collaboration and capacity building [28]. Using these metrics, our findings suggest that the CKD-CP is feasible for use as a point-of-care tool in the primary care setting.

In terms of reach, the CKD-CP is an unrestricted online tool that has been accessed by individuals from a variety of countries, including those in North America and abroad, demonstrating geographical breadth. Previous research has indicated that reminders, interactive small group meetings and mass media campaigns are the most effective dissemination strategies, with unknown effects of social media and networks [23, 29]. We will continue to do multi-faceted dissemination activities, including a prompt (i.e. hyperlink to the website via laboratory reports within Alberta), advertising the pathway on other websites that primary care providers’ access, podcasts and interactive small group discussions with primary care networks.

There was preliminary evidence of CKD-CP usefulness, as determined by end-user satisfaction and perceived value of the pathway based on data from the interviews and the online survey. The CKD-CP was designed for use at the point-of-care, with the ability to diagnose CKD within 3 min (a priority as identified by our primary care stakeholders). On average website users are able to complete a diagnosis in 1.04 min using the *diagnose page* to input lab values via dropdown menus. Our data suggests that the majority of users are proceeding from the diagnose page to the medical management page indicating that users are not leaving the website after using the *diagnose page*. While the average time spent on the *referral page* was 1.29 min, integration of the CKD-CP into the electronic medical record with auto-populating fields for diagnosis and referral could optimize work efficiency in the future. Importantly, the ability to access the tool by members of the primary care multi-disciplinary team was identified as a positive feature as it assisted in clinic workflow processes. This is supported by a recent systematic review which reported improvements in diabetes care for tools that were integrated and available at point-of-care [30]. Our results also reveal that 33 % of users are returning to use the tool for diagnosis, medical management, referral and resources, which suggests that it meets their expectations and needs for caring for patients with CKD.

Some technical limitations were identified. Clinicians using an older browser version experienced limited tool functionality. In Canada, there has been a decrease in use of these older browsers, with only 3 % of computer users between November 2014 and July 2015 using Internet Explorer 8 [31]. The tool was developed to function on newer browsers running on both desktop computers and tablets to enable use of novel interactive features. Technical issues are common with complex software and their use in physician practices [32]. Important strengths of the CKD-CP are its use of evidence-based guidelines to inform the content, as well as its endorsement by academic institutions and Alberta’s single health region. The original purpose of the CKD-CP was to assist primary care providers to diagnose, manage and appropriately refer patients with CKD. Users have reported that the pathway provides CKD knowledge, and can be used to train and educate students and other team members. Website users have also reported that the tool has increased their confidence, which has led to behavior changes in terms of screening, managing and referral of patients with CKD.

Collaborations and capacity building is reflected in the diverse partnerships that have been created and the sharing of information and expertise at local and national levels, including collaborations with a design firm, IT specialists, electronic medical record vendors, laboratory medicine, primary care networks, other health authorities, renal networks, professional associations, primary care professionals and community clinics. Crisp and colleagues state that partnerships can lead to the two-way flow of knowledge which is needed to plan and implement a health program and build capacity [33]. We will continue information sharing and exchange of expertise with other networks and organizations within Canada. We are promoting evidence-based care for patients with CKD through our dissemination and implementation strategies at the local, national and international levels including activities such as joint presentations (with other jurisdictions), international conferences and open access to the tool.

Our use of a mixed methods methodology allowed us to obtain data on the usability and feasibility of the CKD-CP; however the results should be interpreted in light of study limitations. First, the number of respondents to the survey compared to the number of visitors to the site was very small, and may have resulted in a response bias. Second, selection bias may also be an issue for both interviews and the online survey. The majority of participants interviewed had urban-based practices and their responses may not reflect those of rural primary care providers. Third, our evaluation at this stage is primarily descriptive and ecological in nature. Finally, our presented work does not quantify clinician and system behavior change or clinical and process outcomes per se, but does highlight website awareness, interest and current use of knowledge related to CKD. These findings are important for making refinements to the CKD –CP website via ongoing discussions with clinicians and the development team. Stages 5 and 6 of the Knowledge-To-Action Cycle; evaluating process and clinical outcomes and sustaining knowledge use will be the focus of a forthcoming publication.

## Conclusions

Using the Knowledge-To-Action Cycle as a framework, we report the development and implementation of an online adult CKD-CP. Our findings highlight the importance of assessment of end-users awareness, satisfaction with the content, accessibility and perceived quality of the pathway. The CKD-CP was designed to address the evidence to practice gap in the care of patients with CKD identified in our previous work: time limitations; knowledge; and communication with specialists. Our findings have identified future end-user and stakeholder needs to ensure sustainability, including continual dissemination, additional patient materials, electronic referral capabilities and integration into electronic medical records.

## Additional file

Additional file 1: A) Diagnose page B) Medical Management page C) Referral page D) Resources page. (PDF 15822 kb)

## Abbreviations

ACEi/ARB: Angiotensin converting enzyme inhibitor or angiotensin receptor blocker
ACR: Random urine albumin-creatinine ratio
CKD: Chronic kidney disease
CKD-CP: Chronic kidney disease clinical pathway
eGFR: Estimated glomerular filtration rate
N: Nurse
NEPH: Nephrologist
PCP: Primary care physician
PHARM: Pharmacist
