# Supplementary material for: Development and implementation of an online clinical pathway for adult chronic kidney disease in primary care: a mixed methods study
Source: BMC Med Inform Decis Mak. 2016 Aug 17;16:109. doi: 10.1186/s12911-016-0350-z (PMC4989366; doi:10.1186/s12911-016-0350-z)
Supplement: Additional file 1: — A) Diagnose page B) Medical Management page C) Referral page D) Resources page. (PDF 15822 kb) [file 12911_2016_350_MOESM1_ESM.pdf]

A)

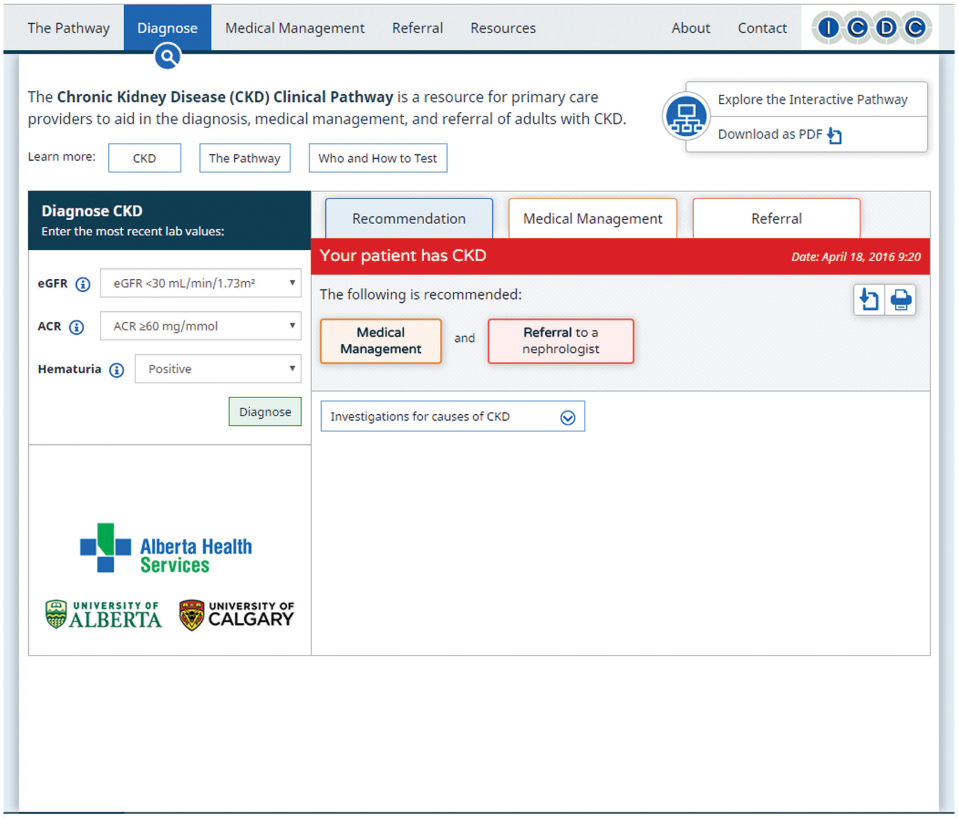

The Pathway
Diagnose
Medical Management
Referral
Resources
About
Contact
I C D C
The Chronic Kidney Disease (CKD) Clinical Pathway is a resource for primary care providers to aid in the diagnosis, medical management, and referral of adults with CKD.
Learn more:
CKD
The Pathway
Who and How to Test
Explore the Interactive Pathway
Download as PDF
Diagnose CKD
Enter the most recent lab values:
eGFR
eGFR <30 mL/min/1.73m²
ACR
ACR ≥60 mg/mmol
Hematuria
Positive
Diagnose
Recommendation
Medical Management
Referral
Your patient has CKD
Date: April 18, 2016 9:20
The following is recommended:
Medical Management
and
Referral to a nephrologist
Investigations for causes of CKD
Alberta Health Services
UNIVERSITY OF ALBERTA
UNIVERSITY OF CALGARY

B)

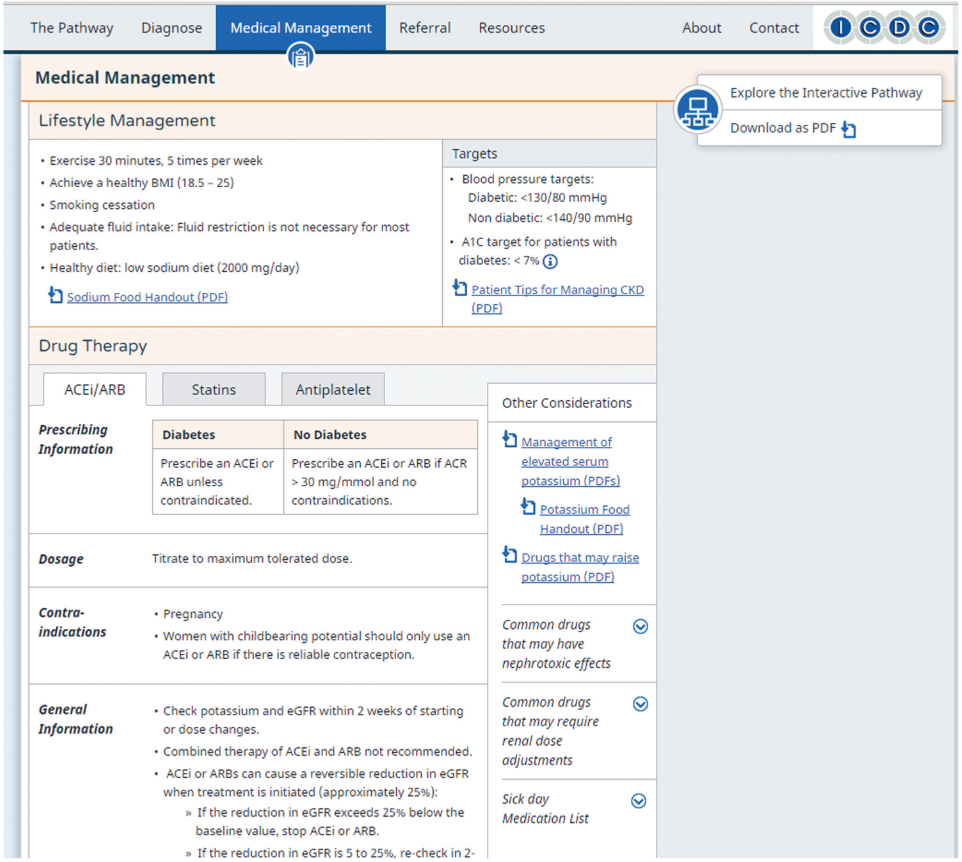

The Pathway
Diagnose
Medical Management
Referral
Resources
About
Contact
I C D C
Medical Management
Explore the Interactive Pathway
Download as PDF
Lifestyle Management
• Exercise 30 minutes, 5 times per week
• Achieve a healthy BMI (18.5 – 25)
• Smoking cessation
• Adequate fluid intake: Fluid restriction is not necessary for most patients.
• Healthy diet: low sodium diet (2000 mg/day)
Sodium Food Handout (PDF)
Targets
• Blood pressure targets:
Diabetic: <130/80 mmHg
Non diabetic: <140/90 mmHg
• A1C target for patients with diabetes: < 7%
Patient Tips for Managing CKD (PDF)
Drug Therapy
ACEi/ARB
Statins
Antiplatelet
Prescribing Information
Diabetes
No Diabetes
Prescribe an ACEi or ARB unless contraindicated.
Prescribe an ACEi or ARB if ACR > 30 mmol/mmol and no contraindications.
Dosage
Titrate to maximum tolerated dose.
Contra-indications
• Pregnancy
• Women with childbearing potential should only use an ACEi or ARB if there is reliable contraception.
General Information
• Check potassium and eGFR within 2 weeks of starting or dose changes.
• Combined therapy of ACEi and ARB not recommended.
• ACEi or ARBs can cause a reversible reduction in eGFR when treatment is initiated (approximately 25%):
» If the reduction in eGFR exceeds 25% below the baseline value, stop ACEi or ARB.
» If the reduction in eGFR is 5 to 25%, re-check in 2-
Other Considerations
Management of elevated serum potassium (PDFs)
Potassium Food Handout (PDF)
Drugs that may raise potassium (PDF)
Common drugs that may have nephrotoxic effects
Common drugs that may require renal dose adjustments
Sick day Medication List

C)

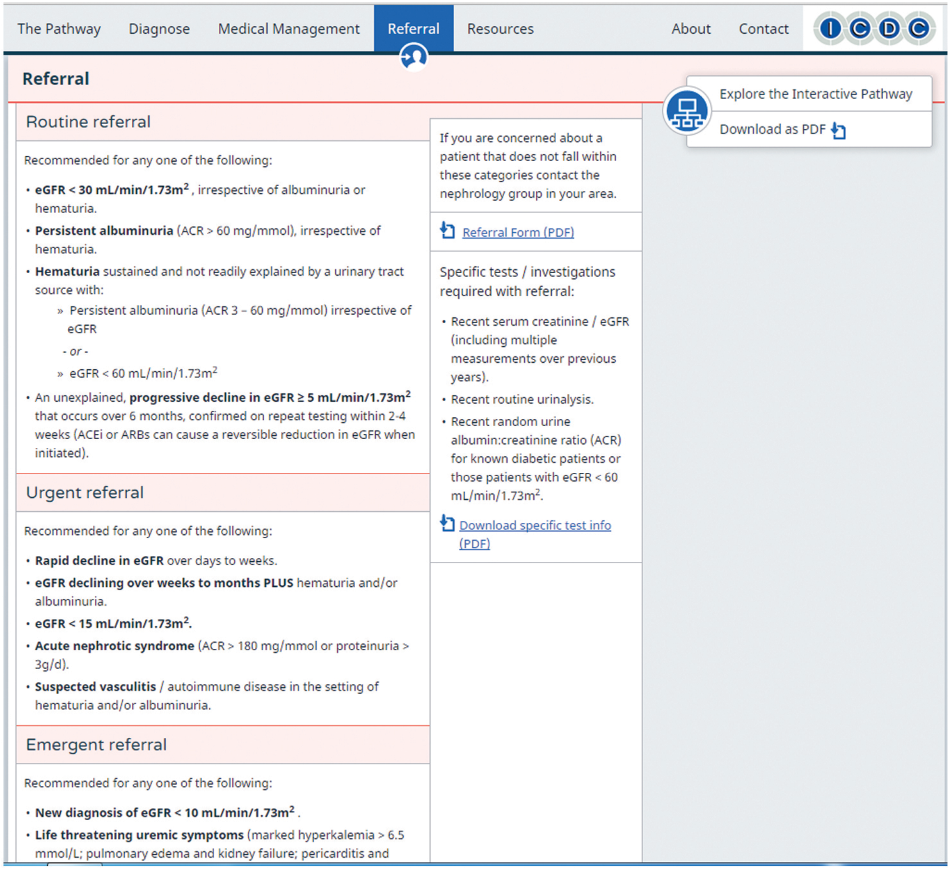

The Pathway | Diagnose | Medical Management | Referral | Resources | About | Contact | ICDC

Referral

Explore the Interactive Pathway

Download as PDF

Routine referral

Recommended for any one of the following:

- **eGFR < 30 mL/min/1.73m^2** , irrespective of albuminuria or hematuria.
- **Persistent albuminuria** (ACR > 60 mg/mmol), irrespective of hematuria.
- **Hematuria** sustained and not readily explained by a urinary tract source with:
  - » Persistent albuminuria (ACR 3 – 60 mg/mmol) irrespective of eGFR
  - *- or -*
  - » eGFR < 60 mL/min/1.73m^2
- An unexplained, **progressive decline in eGFR ≥ 5 mL/min/1.73m^2** that occurs over 6 months, confirmed on repeat testing within 2-4 weeks (ACEi or ARBs can cause a reversible reduction in eGFR when initiated).

If you are concerned about a patient that does not fall within these categories contact the nephrology group in your area.

Referral Form (PDF)

Specific tests / investigations required with referral:

- Recent serum creatinine / eGFR (including multiple measurements over previous years).
- Recent routine urinalysis.
- Recent random urine albumin:creatinine ratio (ACR) for known diabetic patients or those patients with eGFR < 60 mL/min/1.73m^2.

Download specific test info (PDF)

Urgent referral

Recommended for any one of the following:

- **Rapid decline in eGFR** over days to weeks.
- **eGFR declining over weeks to months PLUS** hematuria and/or albuminuria.
- **eGFR < 15 mL/min/1.73m^2.**
- **Acute nephrotic syndrome** (ACR > 180 mg/mmol or proteinuria > 3g/d).
- **Suspected vasculitis** / autoimmune disease in the setting of hematuria and/or albuminuria.

Emergent referral

Recommended for any one of the following:

- **New diagnosis of eGFR < 10 mL/min/1.73m^2** .
- **Life threatening uremic symptoms** (marked hyperkalemia > 6.5 mmol/L; pulmonary edema and kidney failure; pericarditis and

D)

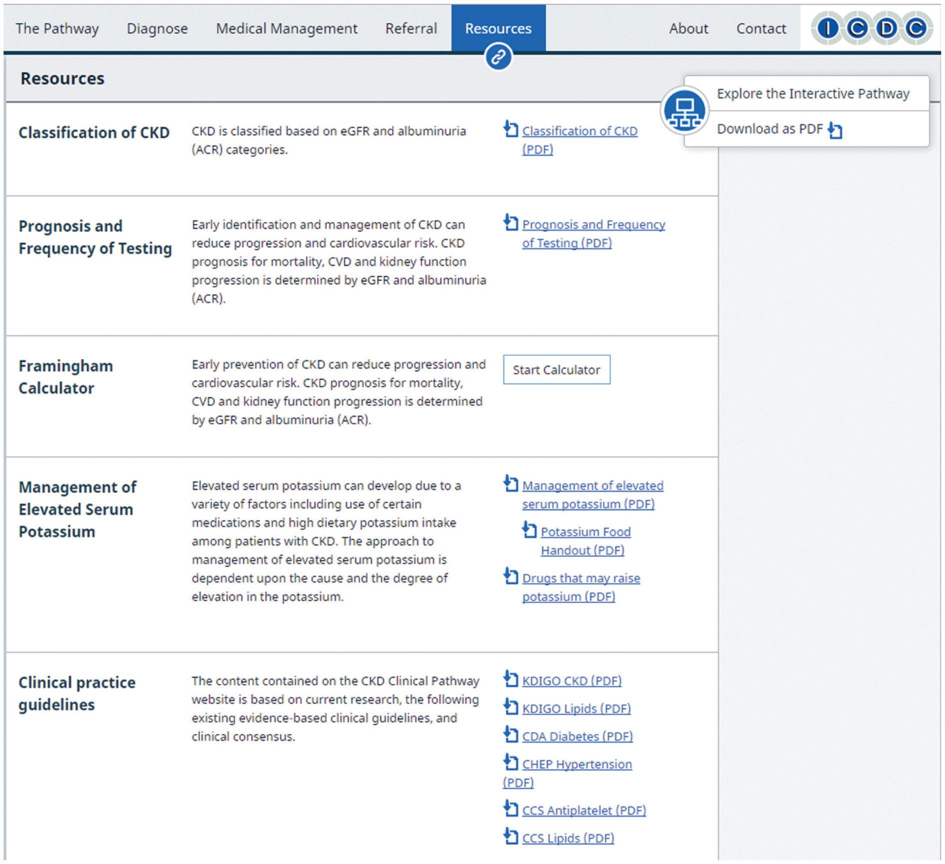

The Pathway | Diagnose | Medical Management | Referral | Resources | About | Contact | ICDC

Resources

Explore the Interactive Pathway

Download as PDF

**Classification of CKD**	CKD is classified based on eGFR and albuminuria (ACR) categories.	Classification of CKD (PDF)
**Prognosis and Frequency of Testing**	Early identification and management of CKD can reduce progression and cardiovascular risk. CKD prognosis for mortality, CVD and kidney function progression is determined by eGFR and albuminuria (ACR).	Prognosis and Frequency of Testing (PDF)
**Framingham Calculator**	Early prevention of CKD can reduce progression and cardiovascular risk. CKD prognosis for mortality, CVD and kidney function progression is determined by eGFR and albuminuria (ACR).	Start Calculator
**Management of Elevated Serum Potassium**	Elevated serum potassium can develop due to a variety of factors including use of certain medications and high dietary potassium intake among patients with CKD. The approach to management of elevated serum potassium is dependent upon the cause and the degree of elevation in the potassium.	Management of elevated serum potassium (PDF)<br>Potassium Food Handout (PDF)<br>Drugs that may raise potassium (PDF)
**Clinical practice guidelines**	The content contained on the CKD Clinical Pathway website is based on current research, the following existing evidence-based clinical guidelines, and clinical consensus.	KDIGO CKD (PDF)<br>KDIGO Lipids (PDF)<br>CDA Diabetes (PDF)<br>CHEP Hypertension (PDF)<br>CCS Antiplatelet (PDF)<br>CCS Lipids (PDF)
